# The Value of SSTR2 Receptor-Targeted PET/CT in Proton Irradiation of Grade I Meningioma

**DOI:** 10.3390/cancers13184707

**Published:** 2021-09-20

**Authors:** Maciej J. Pelak, Birgit Flechl, Marta Mumot, Razvan Galalae, Slavisa Tubin, Eugen Hug, Carola Lütgendorf-Caucig

**Affiliations:** 1 MedAustron Ion Therapy Center, 2700 Wiener Neustadt, Austria; birgit.flechl@medaustron.at (B.F.); marta.mumot@medaustron.at (M.M.); razvan.galalae@medaustron.at (R.G.); slavisa.tubin@medaustron.at (S.T.); Eugen.hug@medaustron.at (E.H.); carola.luetgendorf-caucig@medaustron.at (C.L.-C.); 2 Medizinische Fakultät, Christian-Albrechts-Universität zu Kiel, 24118 Kiel, Germany

**Keywords:** meningioma, PET/CT, DOTA, proton therapy

## Abstract

**Simple Summary:**

Meningiomas are the most frequent tumors of the central nervous system. Their treatment outcomes depend on the histologic grade. Grade I tumors, even bulky and unresectable, are well curable with radiation; therefore, most precise and conservative techniques such as proton therapy are utilized. To better distinguish between tumor extent and normal tissues, PET/CT using tracers that bind specifically to somatostatin receptor type 2 (SSTR2), commonly expressed by these tumors, can be used in addition to MRI. In an experimental blinded study of 30 pre-operated WHO grade I meningiomas independently delineated by four radiation oncologists, we confirmed that the overwhelming majority of meningiomas do express SSTR2 and that the addition of receptor-targeted PET/CT helps visualize lesions not unanimous in MRI and improves the homogeneity of tumor volume definition between observers. SSTR2-targeted PET/CT should be a standard in the planning of curative radiation in pre-operated meningioma.

**Abstract:**

Grade I meningioma is the most common intracranial tumor in adults. The standard imaging for its radiation treatment planning is MRI, and [^68^Ga]1,4,7,10-tetraazacyclododecane-1,4,7,10-tetraacetic acid (DOTA)-conjugated PET/CT can further improve delineation. We investigated the impact of PET/CT on interobserver variability in identifying the tumor in 30 anonymized patients. Four radiation oncologists independently contoured residual tumor volume, first using only MRI and subsequently with the addition of PET/CT. Conformity indices (CIs) were calculated between common volumes, observer pairs and compared to the volumes previously used. Overall, 29/30 tumors (96.6%) showed [^68^Ga]Ga-DOTA avidity. With help of PET/CT, the participants identified six cases with new lesions not recognized in MRI, including two where new findings would critically alter the target volume used for radiation. The PET/CT-aided series demonstrated superior conformity, as compared to MRI-only between observer pairs (median CI = 0.58 vs. 0.49; *p* = 0.002), common volumes (CI = 0.34; vs. 0.29; *p* = 0.002) and matched better the reference volumes actually used for patient treatment (CI = 0.55 vs. 0.39; *p* = 0.008). Cis in the PET/CT-aided series were lower for meningiomas outside of the skull base (0.2 vs. 0.44; *p* = 0.03). We conclude that SSTR2 receptor-targeted PET/CT is a valuable tool for planning particle therapy of incompletely resected meningioma. It serves both as a workup procedure and an aid for delineation process that reduces the likelihood of marginal misses.

## 1. Introduction

Meningioma, a tumor originating from the cells of meninges, is the most common intracranial neoplasm in humans. Treatment of meningioma is a multidisciplinary procedure that is primarily dependent upon histologic grade and symptoms. The most common WHO grade I tumors that make up for 80% of all meningiomas [1] are classified as benign, and due to very slow typical growth pace, a watchful waiting strategy can be successfully applied for tumors that do not cause or threaten with symptoms. On the other hand, for symptomatic grade I meningiomas, surgical resection is the therapy of choice [2]. Inoperable or incompletely resected tumors that are either progressive, symptomatic, or likely to become due symptomatic should receive definite irradiation. While only a small percentage of patients achieve tumor shrinkage, radiation is reported to be extremely effective in achieving disease stabilization, and long patient survivals after the treatment are common [3].

Due to this particularly favorable prognosis, the use of modern radiation techniques that are effective in achieving both tumor coverage and optimal normal organ sparing seems justified. Radiosurgery and oligofractionated radiation found their application in meningiomas of small and spheroid-like volume [4]. For meningiomas of the skull base, the treatment needs to be delivered across 5–6 weeks, and due to complex geometric tumor contour in these patients, proton beam therapy (PBT) appears to be the most optimal radiation modality. Due to its unique physical beam characteristics (entrance dose 20–30% of the maximum, precisely setting each beam’s maximum dose at the tumor depth, no exit dose), it demonstrates the ability to accurately shape the required radiation dose around the tumor [5] and has been successfully explored in the treatment of meningioma [6].

**Figure 1:**
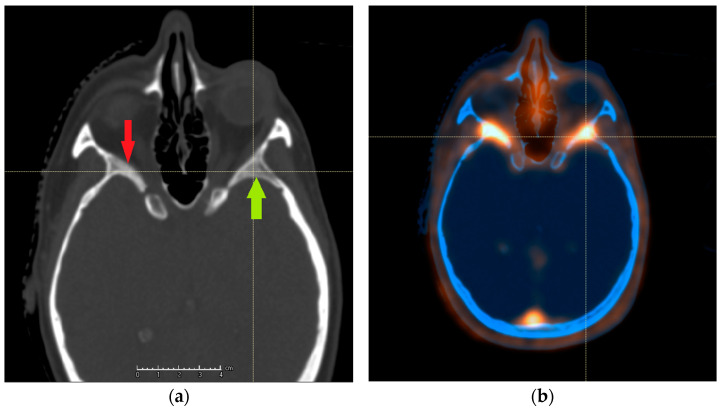
Example of an intraosseous extension of meningioma not properly recognized on CT: (**a**) the right side displays suspicious hyperostosis (red arrow), and the left side appears to have the normal bone structure preserved (green arrow). PET/CT revealed meningioma extension on both sides (**b**).

A crucial step in the application of such a highly conformal radiation technique is the proper definition of tumor extent. The standard aid to native planning CT is the contrast-enhanced MRI, which has the ability to differentiate between soft tissues including meninges superior to the one of CT. In a postoperative situation, its interpretation can be difficult, as scar tissue and normal meninges take up contrast; moreover, rare but known intraosseous and soft tissue extensions could also be either overlooked or overdiagnosed [7,8]. An example of an intraosseous lesion is shown in Figure 1. Meningiomas have been reported to commonly overexpress somatostatin receptor type 2 (SSTR2), and this property can be used to back up structural imaging with a biological study [9]. Multiple groups reported an improvement in tumor extent recognition by using 1,4,7,10-tetraazacyclododecane-1,4,7,10-tetraacetic acid (DOTA)-conjugated PET/CT, yet the available studies featured mixed groups of different meningioma grades and patients treated with different modalities [10,11,12,13]. We, therefore, decided to investigate the impact of DOTA PET/CT on interobserver variability in contouring of meningiomas treated with a uniform protocol and assess the possible clinical consequences.

## 2. Materials and Methods

### 2.1. Patient Selection

After receiving approval from the regional ethics committee, we identified 30 patients treated with a uniform protocol for intracranial meningioma. All patients had to meet the following inclusion criteria: (1) histologically confirmed WHO grade I meningioma; (2) status post at least one surgery with a suspicion of macroscopic residual tumor based on either surgical protocol or postoperative imaging; (3) no contraindications for performing [^68^Ga]Ga-DOTA PET/CT; (4) completion of definitive proton therapy 12 or more months prior to the study inclusion. Relevant patient characteristics are presented in Table 1.

### 2.2. Imaging Protocols

Preoperative MRT images were acquired from multiple external institutions; all of them included at least one T2-weighted, T1-weighted, and T1 + contrast medium sequence. All postoperative MRIs for proton therapy planning were performed using the standardized protocol of our institution. The following sequences were acquired on Philips Ingenia 3T with a standard head coil: T1-weighted (3D mode, 768 × 768 px resolution, 4 mm slice thickness), T2-weighted (3D, 512 × 512, 1.9 mm), T1-weighted with contrast medium (3D, 864 × 864, 0.95 mm), fluid-attenuated inversion recovery (FLAIR, 560 × 560, 4 mm), diffusion-weighted images (DWI) in turbo-spin echo, b = 0–1000 s/m^2^, and apparent diffusion coefficient (ADC, both 256 × 256, 4 mm).

PET/CT studies were performed at least 6 weeks after last surgery in external nuclear medicine departments with one of the three available SSTR2-avid radiotracers: [^68^Ga]Ga-DOTATOC, [^68^Ga]Ga-DOTANOC, or [^68^Ga]Ga-DOTATATE. After fasting for at least 6 h, the patients were intravenously administered 75–273 MBq of tracer activity. After a resting period of up to 60 min, acquisition of low-dose CT and, subsequently, attenuation-corrected PET within 4 to 45 min (according to protocols of external institutions) of the head followed. The maximum standard uptake values (SUV_max_) and SUV_max_-to-background ratio, defined as SUV_max_ of the tumor lesion divided by SUV_max_ of a representative region of interest (ROI) placed in the adjacent meninges or soft tissue, were recorded using IntelliSpace 8 software (Philips, Eindhoven, The Netherlands).

The MRI and PET/CT images were transferred to our treatment planning system (Raystation 8, RaySearch Labs, Stockholm, Sweden) and appended to the patient cases with rigid registration matched on the skull. The anonymized imaging datasets containing preoperative and planning contrast-enhanced MRIs were independently presented to 4 radiation oncology specialists of various experience and expertise from our department. In the first part of the study, the participants were asked to contour the macroscopic residual tumor volume based on available imaging. After all cases were delineated, the contours were locked and PET/CT series were appended to the cases. Participants were subsequently asked to create a copy of their previously contoured volume and adapt, if required, after taking the additional information of the PET/CT series into account. Consulting other colleagues or radiologists was not allowed at any stage, and the possibility of cross-comparison of the contoured volumes was barred in the planning system. In the final part of the study, the original target volumes used for planning the actual radiation treatment (later referred to as “reference volumes”) were reuploaded.

### 2.3. Proton Treatment Protocol

For all patients, 2 cone-down clinical target volumes (CTVs) were used: a smaller high-dose CTV2 defined as macroscopic tumor volume and CTV1, which corresponded to CTV2 with an additional 5 mm margin for possible microscopic tumor spread along the meninx and soft tissues in contact with the tumor. For planning target volumes (PTVs), a 3 mm margin for setup inaccuracy and intrafractional motion was added to both CTVs according to our institution’s protocol. The surgical access path, as well as brain parenchyma and compressed structures not suspicious for infiltration, were excluded from the CTVs. The prescription dose was standard for all patients: 54 Gy of relative biological efficacy (RBE) for PTV2 and 50.4 Gy RBE for PTV1 applied using simultaneous integrated boost technique in 27–30 fractions. The required target volume coverage standard was to achieve a median dose to PTVs equal to the prescribed dose, and that 100% of target volumes were covered in respective 95% isodoses [14]. According to internal and international quality assurance standards, both target volumes and final treatment plans were presented and discussed on internal review boards prior to handover to the medical physicists for planning and before the treatment start, respectively. PBT was applied once per day, Monday to Friday, using the spot scanning technique only.

### 2.4. Statistical Analysis

To assess conformality between volumes, conformality indices (CIs) were calculated. These were defined as the ratio between intersection (common volume) and union (summary volume), which for hypothetical, ideally conform volumes would equal 1. The respective volumes were created in Raystation 8 using the in-built algebra function. CIs were calculated for MRI-only and PET/CT-assisted series separately between each possible pair of observers, between each observer and reference volume, and between intersection volume of all participants and reference volume. The statistical calculations were carried out using StataIC 15 (StatSoft, Tulsa, OK, USA). The analysis of differences between the series was assessed by non-parametric Wilcoxon matched-pairs signed-rank test. The test was two sided, and results with *p*-value below 0.05 were considered statistically significant. Cases of most critical deviation between corresponding target volumes with and without the aid of PET/CT were then selected for dosimetric evaluation.

## 3. Results

### 3.1. PET/CT Findings

All tumors, except for one, presented a clearly increased radiotracer uptake (median SUV_max_ = 13.5; range: 6.6–31.2) and a high-contrasting tumor-to-background ratio (median 8.2:1; range 3.7:1–20.8:1). The single non-avid case (SUV_max_ = 2.0, contrast ratio ~1:1) was excluded from further analysis. In 6 (20.6%) of the remaining 29 cases, PET/CT revealed new findings not unanimously identifiable in MRI-only based assessment. Three of these (10.3%) corresponded to additional small peripheral meningiomas of the falx, and in one case, the new finding was an asymptomatic intraosseous extension in a patient irradiated for a meningioma of the falx. In all actual cases, these were interpreted as coincidental findings distant from the actual target volume, and their management was concluded to be watchful waiting with resection or irradiation seeming feasible in case of radiologic and/or symptomatic progression.

The other 2 lesions not identifiable on MRI but correctly delineated by all participants in PET/CT-aided series were either in direct continuation of the MRI-only target volume or in direct proximity. These included an uncharacteristic intraosseous extension in the sphenoid and an intraorbital infiltration of the medial rectus muscle. In clinical assessment, they were regarded as unlikely to be completely resectable in the future and therefore should have been included in the irradiated volume.

### 3.2. Conformity Analysis

In the volumetric analysis, we could observe a trend towards a slight increase of contoured tumor volume after re-delineating with PET/CT imaging (increase in 24 cases out of 29, median absolute/relative volume change for the common volume of all observers = +1.77 cm^3^/+20.2%). The contours delineated in PET/CT-aided series were significantly more uniform between study participants: both when analyzed between all possible observer pairs (median CI = 0.486; range: 0.16–0.72 for MRI-only and 0.575; range: 0.332–0.781 for PET/CT-assisted series; *p* = 0.002) and when compared with contour intersections from all observers between the series (respectively, CI = 0.29; range: 0.02–0.54 and median CI = 0.34; range: 0.09–0.64; *p* = 0.002). In both analyses, CIs were more frequently higher for PET/CT-assisted contours (23/29, 79.3%) than for MRI-only (6/29, 20.7%). It is worth mentioning that three out of six cases with higher CIs in MRI-only based series were ones in which new findings, not recognized on MRI, were identified by PET/CT by all study participants. We also identified tumor location other than skull base to feature lower Cis, compared to meningiomas of the central skull base location within the PET/CT-assisted series (median 0.2 vs. 0.44; *p* = 0.03).

In the analysis of conformity between the intersection of participants’ contours and the reference target volumes, a statistically significant difference was observed which, in line with the previous observation, indicated a higher median CI between reference contours and PET/CT assisted series (0.39 MRI-only vs. 0.55 PET/CT-assisted; range: 0–0.779 and 0.07–0.772; *p* = 0.008).

**Figure 2:**
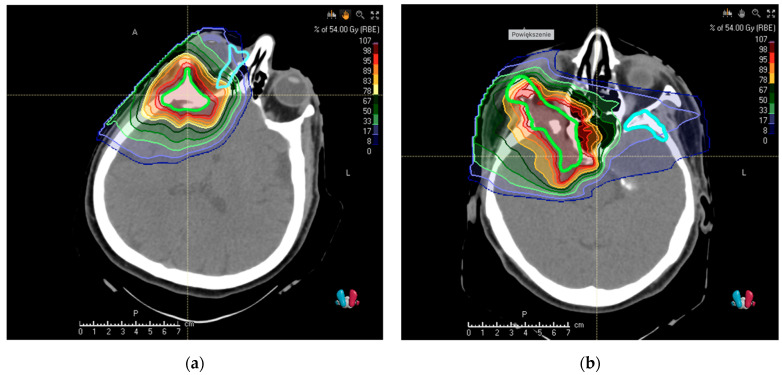
Display of dosimetric consequences of omission of tumor volume in two cases in which PET/CT delivered new crucial information in addition to CT + MRI. Thick fluorescent contours represent volumes of observers performed using MRI only, thick light blue contours display the newly identified tumor extent: (**a**) intraorbital (**b**) in the left sphenoid bone. The treatment plans were carried out for MRI volumes only.

For depicting dosimetric consequences of target volume change after recontouring with PET/CT, we selected patients with the newly found critical tumor extension. New PBT treatment plans according to the protocol described in the Methods Section were created for target volumes performed by using MRI only; PET/CT-assisted volumes were subsequently appended to display the possible underdosage. These are displayed in Figure 2a,b; the relevant dosimetric statistics are displayed in Table 2.

## 4. Discussion

The development of highly conformal techniques in radiation oncology seems promising for sparing the healthy organs at risk in proximity to the tumor but requires a precise target volume delineation. Due to high dose falloff within millimeters, a high level of certainty is required whether an abnormal or asymmetric tissue corresponds to the residual tumor or post-therapeutic lesion. Routine use of multiparametric MRI improves this process, yet the idea of backing up structural imaging with one that uses unique biological properties of the tumors is increasing in significance. PET/CT or PET/MRI targeting receptors that are explicitly overexpressed in tumor tissue is particularly promising, as very high contrast and specificity can be expected. This was confirmed in our study, which found an overwhelming majority of the tumors to be well distinguishable from the adjacent tissue, with a high average 8:1 contrast ratio and no tumors omitted in PET/CT-aided delineations. Experience and interaction with nuclear medicine specialists are required to properly interpret areas of low uptake; in the case of DOTA PET/CT, these can be attributable to developing scar or residual inflammation—fibroblasts and other immunogenic cells do express SSTR2 when exposed to pro-inflammatory stimuli [15]. Rachinger et al. correlated presurgical PET/CT scans of meningiomas and normal tissue. They identified SUV of 2.3 as the tumor/non-tumor cutoff value and increased sensitivity of PET/CT delineation, compared to MRI-only assessment with specificity being comparable [16]. Both findings are in line with our results, as the average SUV_max_ value of a representative adjacent tissue was 1.6 in our study. We identified intraosseous meningioma extension as particularly prone to the omission in MRI-based contouring that could lead to major deviations from optimal tumor coverage. A study by Kunz et al. on transosseous meningiomas confirms the added value of DOTA PET/CT in identifying these concerning areas of tumor volume, with sensitivity increasing from 53.7%, for MRI-only based assessment, to a highly confident 98.5% when additionally using PET/CT [17].

Studies of different authors commonly report a meaningful alteration in size of the contoured volume in 65–77% of patients [10,11] after backing up MRI assessment with DOTA PET/CT. Data of Graf et al., who also evaluated median absolute and relative change in size between MRI-only and PET/CT-assisted series, identified values very similar to our results: 1.5 cm^3^ average absolute size increase attributable to PET/CT findings (16–34% relative). Our study, apart from focusing on standardized patient groups treated with uniform modality, was one of the two to assess in detail the conformity between multiple observers in DOTA PET/CT for meningioma. The results of Maclean et al. indicated a minor improvement of CIs for CTV between observers (0.35 vs. 0.31; *p* = 0.04) in a group of 10 patients [18]. Stade et al. performed an interesting analysis on the potential impact of contouring variability on radiation doses to adjacent organs at risk. They analyzed this influence separately for photon and proton-based radiation for 10 patients, and interestingly, significant differences in doses to OARs were only confirmed for photon irradiation. This finding confirms the very high OAR sparing capability of proton therapy and, accounting for the exceptionally high curability of meningioma using PBT, indicates that the primary goal of using DOTA PET/CT for treatment planning of meningioma in this modality would be avoiding marginal misses on tumor areas difficult to identify in MRI. This would be of particular importance in patients after multiple surgeries or previous irradiation, in whom a whole spectrum of post-therapeutic findings can mimic actual tumor extent [19].

The usability of DOTA PET/CT for therapy planning in meningiomas raises questions whether this modality should be also considered for patient follow-up, in particular, because of the very limited number of such studies for receptor-based PET tracers [20]. Due to the response pattern of WHO grade I meningioma for radiation treatment (excellent local control but in an overwhelming majority of cases corresponding to stable disease), this appears controversial; therefore, follow-up PET/CT with ^68^Ga-tracers would be likely indicated only in case of diagnostic doubts in MRI. For WHO grade II-III tumors featuring significantly increased risk of recurrence, compared to grade I, it seems feasible to perform an experimental study with routine follow-up PET/CT to identify whether tracer accumulation pattern in subsequent examinations can predict recurrence risk. The results might give an insight into the biological basis of meningioma recurrences, as it remains unanswered whether the dedifferentiation phenomenon (reflected by loss of receptor expression) affects the prognosis, similar to, e.g., neuroendocrine tumors [21].

A number of limitations of our study need to be mentioned. As our institution relies on external PET/CT studies, the variabilities in examination protocols (the use of multiple tracers that have different SSTR2 affinities, tracer doses, and acquisition times) could possibly influence the interpretation of the studies, in particular concerning areas of moderate ^68^Ga-tracers uptake. Additionally, for this reason, the SUV values presented in our results should be interpreted carefully. More frequent use of [^68^Ga]Ga-DOTATATE, which features higher receptor affinity, could possibly further improve the tumor-to-background ratio. The observers of our study all had previous and current experience of meningioma treatment using PBT, yet certain individually learned approaches could contribute to variability in defining the gross tumor volume where a decision whether not to include a certain finding in the target volume is possibly more difficult to make than inclusion. This has to be addressed by precisely defining the contouring standards within the institution and also setting up standards of gross tumor volume definition. Multidisciplinary analysis of multiple cases and referring to studies that correlate quantitative imaging findings with surgical specimens [16] are primary means to support this process.

## 5. Conclusions

SSTR2 receptor-targeted PET/CT is a valuable tool for planning particle therapy of incompletely resected meningioma. On the one hand, it serves as a workup procedure that identifies additional lesions needing further observation or separate treatment options. On the other hand, it increases confidence in the delineation process, helping to reduce the risk of tumor volume omission and achieve uniformity between treating physicians independent of their level of experience and personal preferences. It is particularly recommended for patients who received multiple previous treatments and with tumors in uncommon locations, in whom anatomic conditions and inadequate observer experience may lead to suboptimal target volume definition. Standards of imaging interpretation should be established and actively discussed within the institution to achieve high target volume conformality and, as a final effect, delivery of treatment that is both long-term efficient and safe.

## Figures and Tables

**Table 1:** Patient characteristics.

Variable (Unit)	No. of Patents (%)	Median (Range)
Age (years)		56.5 (28.2–81.1)
No. of surgeries		
1	25 (82.8%)	
>1	5 (17.2%)	
Previous irradiation		
No	27 (89.7%)	
Yes	3 (10.3%)	
Location		
Skull base	24 (80%)	
Olfactory	2 (6.7%)	
Orbit	2 (6.7%)	
Falx	2 (6.7%)	
Radiotracer		
[^68^Ga]Ga-DOTANOC	19 (63.3%)	
[^68^Ga]Ga-DOTATOC	10 (33.3%)	
[^68^Ga]Ga-DOTATATE	1 (3.3%)	

**Table 2:** Dosimetric evaluation of cases with critical new information delivered by [^68^Ga]Ga-DOTA PET/CT.

Case No.	Total Tumor Volume (cm^3^)	Tumor Volume Delineated Using MRI (cm^3^)	Tumor Volume Not Recognized in MRI (cm^3^)	Tumor Volume Not Recognized on MRI (%)	Mean Radiation Dose to the Tumor Volume Omitted in MRI (Gy RBE)	SUV_max_ of the Additional PET Finding
1	34.85	31.81	3.04	8.7	18.51	4.4
2	59.64	52.72	6.92	11.6	11.26	12.98

## Data Availability

The data contain patient-related medical information. They are confidential and only available to certified trial auditors.
